# Investigating Orthopaedic Surgical Prophylaxis Changes and Post-operative Acute Kidney Injury (AKI) in NHS Grampian - a Test Case for NHS Scotland

**DOI:** 10.23889/ijpds.v1i1.361

**Published:** 2017-04-19

**Authors:** Charis Marwick, Jacqui Sneddon, Eilidh Fletcher, Andrea Patton, Gwen Bayne

**Affiliations:** 1 Population Health Sciences Division, University of Dundee; 2 Scottish Antimicrobial Prescribing Group; 3 Information Services Division, NHS National Services Scotland

## Objectives

To support reduction of Clostridium difficile infection (CDI), in 2008 the Scottish Antimicrobial Prescribing Group recommended that all NHS boards in Scotland restrict the use of antibiotics associated with a high risk of CDI. In NHS Grampian the policy for antibiotic prophylaxis in orthopaedic surgery was changed in June 2010. Trauma patients undergoing internal fixation or arthroplasty received flucloxacillin or co-amoxiclav before the policy change and flucloxacillin and gentamicin after the policy change. Previous studies have found similar policy changes resulted in an increase in post-operative acute kidney injury (AKI) and this study examined rates of post-operative AKI before and after this policy change in the NHS Grampian region of Scotland. Patients undergoing elective arthroplasty received either flucloxacillin or co-amoxiclav or cefuroxime before the policy change and cefuroxime afterwards.

## Approach

All NHS Grampian patients who underwent an orthopaedic surgical procedure with prophylaxis during the period 01 June 2008 to 31 May 2012 were selected. Cases were linked to local creatinine data to detect post-operative AKI. Cases were further linked to national coverage data: (i) hospital discharge data to create the Charlson score for comorbidity, (ii) patient-level community prescribing data to identify previous exposure to any medicines which predispose to renal impairment, (iii) patient-level infection data to identify any post-operative CDI and (iv) Scottish Renal Registry data for case exclusion purposes. Segmented regression analyses of interrupted time series were used to evaluate changes in level and trend associated with the intervention and estimate effect sizes. 

## Results

3,870 trauma cases and 5,475 elective cases were examined. There was a significant increase in AKI rate following the policy change (\beta=0.28; 95%CI, 0.03 to 0.53; p=0.028) in trauma patients, equating to an increase of 0.28 cases of AKI per 100 procedures per month and a relative intervention effect at 24 months of 150% (95% CI 25% to 250%) There was no significant change in AKI rate among elective patients. 

## Conclusion

We found that a change in orthopaedic antibiotic prophylaxis policy in NHS Grampian to flucloxacillin plus gentamcin was associated with an increase in post-operative AKI. This is consistent with observations in other boards and supports the new recommendation away from this policy made by SAPG in 2012. In cohort of trauma patients we found the change in policy was associated with an increase in post-operative AKI rate. 

